# “*It's Just More Acceptable To Be White or Mixed Race and Gay Than Black and Gay”*: The Perceptions and Experiences of Homophobia in St. Lucia

**DOI:** 10.3389/fpsyg.2017.00947

**Published:** 2017-06-19

**Authors:** Jimmy Couzens, Berenice Mahoney, Dean Wilkinson

**Affiliations:** Psychology Department, University of WorcesterWorcester, United Kingdom

**Keywords:** Caribbean, skin color, colorism, homosexuality, homophobia

## Abstract

Lesbian, gay, and bisexual (LGB) individuals come from diverse cultural groups with differing ethnic and racial identities. However, most research on LGB people uses white western samples and studies of Afro-Caribbean diaspora often use Jamaican samples. Thus, the complexity of Afro-Caribbean LGB peoples' experiences of homophobia is largely unknown. The authors' analyses explore experiences of homophobia among LGB people in St. Lucia. Findings indicate issues of skin-shade orientated tolerance, regionalized disparities in levels of tolerance toward LGB people and regionalized *passing* (regionalized sexual identity shifting). Finally, the authors' findings indicate that skin shade identities and regional location influence the psychological health outcomes of homophobia experienced by LGB people in St. Lucia.

## Introduction

Former British Caribbean colonies including Jamaica, Barbados and the Bahamas (Gaskins, 2013) have been the focus of psychological research on sexual orientation and homophobia in the Caribbean region (e.g., Kempadoo, 2004, 2009; Sharpe and Pinto, 2006). However, Caribbean culture is diverse (Hickling et al., 2009) and we know less about the perceptions and experiences of LGB individuals living in the French Antilles and former Dutch and Spanish colonies despite their distinct cultural identities and attitudes to sexual orientation (Kempadoo, 2004, 2009; Sharpe and Pinto, 2006; Gaskins, 2013). This qualitative study focuses on this gap in the literature by exploring the perceptions and experiences of homophobia among lesbian, gay, and bisexual (LGB) individuals living in St Lucia, an Eastern Caribbean Island with a British and French creolized, or *Kwéyòl*, colonial history, culture and language. Homophobia is fear or intolerance toward people who are attracted to others of the same-sex (Remafedi, 2002; Consolacion et al., 2004). This study focuses on the intolerance aspect of homophobia, and considers the meaning of skin complexion and location for the intolerance experienced by St. Lucian LGB people.

## Background

The 28 territories of the Caribbean have a population of over 35 million people (Baldacchino, 2015; International Union for Conservation of Nature, 2016) and estimates suggest that 20% of the population identify themselves as non-heterosexual (McDonald, 2012). However, across the Caribbean region many Islands criminalize homosexual behavior (Human Rights Watch, 2004; Hickling et al., 2009). All homosexual acts are illegal in Trinidad and Tobago and Barbados; and male homosexuality including sodomy and public displays of affection are illegal in Guyana and Jamaica but female homosexuality is not (Human Rights Watch, 2004; Sheller, 2012). Some law enforcement agencies in the region fail to protect LGB individuals from homophobic hate crime; and some law enforcement officers themselves have been involved in harassment and attacks on men and women perceived to be homosexual (Human Rights Watch, 2004; Becker, 2013; Cloonan, 2013; Stanislas, 2013a,b). The impunity of individuals who commit hate crimes against LGB individuals is likely to legitimize stigma, hatred, abuse, and discrimination against LGB individuals in various Caribbean societies (Smith, 2011; Sheller, 2012; Stanislas, 2013a,b). Consequently, many of the region's LGB residents conceal and suppress their sexual identity to prevent social exclusion or criminalization (Stern, 2003; Hickling et al., 2009). Thus, LGB people in the Caribbean have long struggled for social, cultural, and legal acceptance and tolerance (Coates, 2010; Smith, 2011).

The prevalence of homophobia and homophobic abuse in Jamaica and other Caribbean Islands has been linked to high rates of family disownment, homelessness and loneliness within local LGB communities (Bourne et al., 2012). Homophobia has also contributed to some of the mental health issues experienced by LGB individuals in the region including their greater rates of depression, anxiety and substance misuse disorders compared to heterosexuals (King et al., 2006; Addis et al., 2009; White et al., 2010; Milne, 2011; Bourne et al., 2012). A study of stigma and discrimination experienced by homosexual men in Jamaica found that the majority of participants reported family disownment and being “shamed” into dropping out of school (Bourne et al., 2012). Stigma, discrimination, and homophobic violence led many to believe that their lives were less productive and that consequently their psychological health. This included feelings of depression, suicidality, and chronic sadness that they associated with suppressing and concealing their sexuality (Bourne et al., 2012). White et al. (2010) studied the mental health needs of LGB people in Jamaica and found 45% of their sample reported symptoms associated with major depression. Additionally, 69% met the criteria on the SCID-I/NP (Structured Clinical Interview for DSM-IV-TR Axis I Disorders Non-Patient Edition) for ever having experienced one or more Axis I disorders (DSM-IV-TR) in their lifetime. White et al. (2010) attributed these mental health issues to the “high” incidence of homophobic abuse reported by their sample. Over 50% in their study reported experiencing homophobic abuse, such as name-calling, discrimination, violence, threats of violence, and harassment on more than three occasions each month. However, only a quarter reported incidents to the local authorities, and only 10% received counseling for the depression and trauma they perceived these events to cause. Thus, openness about sexual orientation strongly associated with increased incidences of sexuality-related abuse, violence, and harassment leading White et al. (2010) to link sexuality related openness with poorer psychological health.

Beyond Jamaica there is little published peer reviewed academic or other research on Caribbean LGB communities, making it difficult to understand the experiences of LGB individuals outside this Anglophone Island (Brown, 1997; Sharpe and Pinto, 2006; Kempadoo, 2009; Nelson and Melles, 2010). Therefore, many Caribbean nations including St. Lucia lack evidence that could inform policy and practice designed to support the needs of their LGB communities (Gaskins, 2013). A small body of gray literature, particularly on tourism, the law and politics suggests Caribbean nations differ in their acceptance and tolerance toward LGB people. Homophobia is not entirely absent from Caribbean cultures. However, literature indicates that the French and Spanish Speaking Islands are more tolerant of homosexuality than English speaking Islands (e.g., Chevannes and Gayle, 2000; Chevannes, 2001; Reding, 2003; Zimmerman, 2003; Human Rights Watch, 2004; Gorry and Miller, 2005; Kempadoo, 2009; Coates, 2010; Nelson and Melles, 2010; Porter and Prince, 2011; Smith, 2011; Careaga, 2011; Stanislas, 2013a,b; Sáez, 2015). Evidence suggests historical characteristics of nations could underpin differences in homophobia across the Caribbean. Specifically, those that have a relatively stable Anglo-phone colonial history—Islands that remained predominantly under British colonial rule (e.g., Barbados)—appear more homophobic compared to those that remained predominantly under French and Spanish colonial rule (e.g., Guadeloupe and Cuba). Careaga (2011) explains how Islands that remained predominantly under British colonial rule seem “unable to shake off the influence of Victorian morality” (p. 1). This pattern suggests differences in ideological handovers from the regions colonial past have led to differences in homophobia across the Caribbean (Sharpe and Pinto, 2006; Kempadoo, 2009). Moreover, some Caribbean Islands such as St Lucia have a relatively complex colonial past. St. Lucia has a British and French colonial history that has developed into a creolized (mixed) culture and language known locally as *Kwéyòl* (Baker and Jones, 2000; McWhorter, 2000; Paul, 2007; St-Hilaire, 2011). Unlike other Islands, such as Barbados that remained predominantly British during colonial times, St. Lucian culture represents a unique hybrid of its ideological heritage from the region's colonial past and it retains strong French socio-cultural ideologies, customs, and practices (Strazny, 2011). However, as a culture created from a fusion of what seems to be two opposing ideological extremities (French = tolerant of LGB people vs. English = intolerant of LGB people) it is unclear where the St. Lucian *Kwéyòl* culture is positioned in relation to tolerance and acceptance of LGB people.

Given it's deeply complex cultural heritage, St. Lucia presents a sufficiently different cultural context from that considered in previous research in the region. Therefore, considering St. Lucia can be instructive for how we view and understand homophobia in the Caribbean and the psychological impact of homophobia on LGB people. However, two issues complicate this further: the racialization and coloration of homosexuality and homophobia, and the “developed north” vs. the “underdeveloped south.”

### The racialization and coloration of homosexuality and homophobia

Skin-color stratification is the differentiation of people by lightness and darkness of skin-shade and it continues to be a salient feature and socio-psychological issue in Caribbean societies and cultures (St-Hilaire, 2011). Pigmentocracy or socio-cultural hierarchy based on skin-color that systematically provides privilege based on lightness of skin, is central to St Lucian society (Crowley, 1956; Lowenthal, 1972; Potter, 2003; Hickling et al., 2009; St-Hilaire, 2011; Malcolm, 2012; Shirk and Edmond-Poli, 2012). Within a pigmentocractic society colorism, prejudice and discrimination against dark-skinned individuals are found to be at their peak (Gabriel, 2007; Coates, 2010; Bonilla-Silva, 2011; Jablonski, 2012). Thus, pigmentocracy is a major cause of racial prejudice and separatism in St. Lucia (St-Hilaire, 2011). In St. Lucia, and in many Caribbean societies, pigmentocracy has instigated the creation of multiple skin-color identities (Gamman, 1994; Grugel, 1995; Cox, 2002; Charles, 2008; St-Hilaire, 2011). These identities are ascribed different levels of privilege and define behavioral expectations for members of the community (Bouson, 2000; Reddock, 2004; Kruijt, 2005; Rosario, 2011; St-Hilaire, 2011; Cooper, 2012; Flynn, 2012; Shirk and Edmond-Poli, 2012; Breland-Noble et al., 2016).

Within various Black-American and Black-Caribbean communities there is a racialized understanding of “(ab)normal” and “acceptable” sexual behavior for black persons (Fuss, 1995; Napier, 2000; Carbado, 2001; Alexander, 2004; Kornegay, 2004; Hunter, 2005; Silverman, 2005; Ford, 2006, 2008, 2013; Thomas, 2007; Grosch, 2008; Wahab and Plaza, 2009; Das Nair and Thomas, 2012). Numerous communities perceive homosexuality as “something that white people do” and “blacks should not do” (Carbado, 2001, p. 250). Consequently, identifying as LGB clashes with what it means to be *black* within many of these communities (King, 2004). Since the 1960s rap and dancehall music and culture have reified this philosophy and reinforced the belief that homosexuality is an attribute of white ethnicity and western culture (Carbado, 2001). For this reason some scholars describe dancehall and rap music as ideological weaponry that reinforce Caribbean socio-cultural anxieties in the form of colorism and homophobia (Dawe, 2004; Nelson and Melles, 2010; McGinley and Cooper, 2012).

However, we know relatively little about how skin-color intersects with sexuality in St Lucia and elsewhere in the Caribbean and whether it influences levels of tolerance and acceptance toward LGB individuals. Within a culture where homosexuality is perceived as belonging exclusively to white people and western cultures, it is possible that in St. Lucia skin-shade could impact on the level of sexuality related tolerance and hatred experienced by dark and light-skinned LGB individuals. Ford's (2008, 2013) work on the perceptions and experiences of gender roles in African-American communities informs this interpretation and they state that “lighter skinned or ‘pretty’ men are often implicitly connected with metro/homosexuality” (Ford, 2013, p. 31).

The idea that skin-shade could influence levels of tolerance and hatred toward LGB people in St. Lucia fits the pigmentocractic structure of St. Lucian society. It ascribes social identity, status, and privilege by lightness of skin-shade. This could mean that greater tolerance toward light-skinned LGB people is coherent with the ongoing privileges of lighter-skinned individuals and communities. Within this social context dark-skinned (known in *Kwéyòl* as: *Neg*) LGB people could experience greater levels of homophobic discrimination and hatred than their white (known in *Kwéyòl* as: *Bétjé*—white person) and light-skinned (known in *Kwéyòl* as: *Chaben*—brown-skinned female, *Chabin*—brown-skinned male) peers. Consequently, dark-skinned LGB people could also experience poorer psychological health than their lighter-skinned peers do. This interpretation is consistent with the work of Espejo (2008) who explored the experiences of colorism among male homosexual sex-workers in Thailand. Dark-skinned males were found to experience greater levels of discrimination than their lighter-skinned peers and as a result, their dark-skinned participants reported lower levels of self-esteem and self-worth (Espejo, 2008). Disconcertingly, there is little research on the role of skin-shade in experiences of homophobia and associated psychological health and well-being within and across black populations (Harley et al., 2002, 2012). Thus, by exploring how skin-shade impacts on sexuality-related tolerance and thus the well-being of LGB people in St. Lucia, this study will be one of the first of its kind on Black populations.

There are studies about the dual stigma of homophobia and racism in Black-American populations that might inform the possible psychological health and well-being implications of skin-color oriented tolerance in St. Lucia. For example, Szymanski and Gupta's (2009) study of African-American LGB people suggests they internalize their experiences of homophobia and racism leading to depression and depressive distress (also see (Gupta, 2008)). This suggests that the stigma of “skin-color oriented tolerance” that combines homophobia, racism and colorism into one may similarly be internalized and induce feelings of depression and depressive distress in some dark-skinned LGB people. This is also consistent with studies of intersectionality that explore how people experience and deal with overlapping and conflicting identities (Das Nair and Thomas, 2012). In a socio-cultural environment where it is unacceptable for dark-skinned persons to engage in sexual and romantic relationships with individuals of the same-sex, dark-skinned LGB people may experience conflict between identities of being dark-skinned and LGB. Consequently, some dark-skinned LGB people might be found to experience a cognitive dissonance between the openness and self-acceptance of their sexuality (Darry, 1989; Eliason and Beemyn, 1996; Moji et al., 2009). For instance, studies exploring experiences of homophobia in African-American communities have found that many LGB people report accepting their LGB identity but suppressing and concealing their sexual orientation in order to sustain a positive relationship with their family and community (Battle and Crum, 2007). Similarly, some dark-skinned LGB people might prioritize their dark-skinned identity, and suppress or conceal their LGB identity to sustain positive social relationships. This evidences what might be a cognitive dissonance between the awareness, acceptance, and openness of their sexuality. However, by denying and concealing sexual identity LGB people risk experiencing psychological health problems such as depression that are secondary to the suppression of their *true* self, sexual identity, feelings, and desires (Kanel and Horn-Mallers, 2015). Sexual orientation concealment is also associated with chronic stress and anxiety, more day-to-day worry about others discovering one's true sexual orientation, and having to maintain deceptions used to conceal one's sexuality (Meyer, 2003; Smith and Ingram, 2004).

### The “developed north” vs. the “underdeveloped south”

Over the past two decades, scholars have reported the existence of cultural and economic disparities between the Northern and Southern region of St. Lucia. Southern communities are perceived as more poverty-stricken, traditional in their values and less educated than are communities in the North (Antoine, 1998; Walcott, 1999; St-Hilaire, 2011; St. Lucia News Online, 2016). Socially and in academia the South is regarded as functioning using an outdated way of life and mind-set reflective of ideologies and values originating from British colonial rule (Antoine, 1998; Walcott, 1999; St-Hilaire, 2011). Although, Southern communities increasingly aspire to become more educated and modernized, there is still a lack of social integration between the North and South of the Island and a lack of capital investment in the Southern districts that might impede the Souths ability to achieve reform (Dabydeen, 1988; Gamman, 1994; Walcott, 1999; St-Hilaire, 2011). In February 2016, Dr. Kenny Anthony, while Prime Minister of St. Lucia, directly acknowledged the educational and economic neglect of the South and vowed to create an equal capital investment initiative to ensure fair treatment of people in St. Lucia wherever they might be. Dr. Kenny Anthony, in a press release through St. Lucia News Online (2016), said:

“it's a message that I have drummed into the National Insurance Corporation; emphasized time and time again that all financial investments should not be located in the North, in the Castries basin; that all workers of this country wherever they are located contribute to the resources of the National Insurance Corporation and they too must be part of the investment initiatives of the corporation.” (p. 1).

Nevertheless, how the North vs. South cultural divide has an impact on perceptions and experiences of homophobia is less clear. For example, if Southern communities are operating with an outdated mind-set reflective of ideologies and practices instilled during British colonial rule, they might have a more “victorianized” understanding of sexuality (Careaga, 2011). If so, they may be less culturally tolerant and more hateful toward LGB people (Zane, 1992; Robinson, 2010; Maritz, 2013). Additionally, the lack of education in Southern communities could foster negative attitudes toward LGB people. Studies from Jamaica show that educated people have more positive attitudes toward homosexuals than their lesser educated peers (Boxill, 2012). Studies have linked the intensity of homophobia in Jamaica to what is thought to be a “victorianized” understanding of sexuality instilled during times of British colonial rule (Careaga, 2011). Consequently, education may be an effective tool for moving “out” of the victorianized understanding of sexuality “to” a more accepting ideology. This is consistent with the well-documented associated between a lack of understanding of sexual and gender minorities and intolerance (Klesse, 2016).

Tourism infrastructure is a determinant of regional development that may also play a crucial role in reinforcing differing levels of tolerance and hatred toward LGB people between Northern and Southern regions. The St. Lucian economy depends primarily on tourism and it accounts for ~89.1% of the GDP (National Accounts, 2016). However, tourism investment and development is much greater in the North than in the South (Novelli, 2006; Velde, 2008). Furthermore, the promotion of travel for pleasure between countries contributes not only to economic growth but also to the interchange of knowledge between citizens that helps promote greater mutual understanding and co-operation (Mings, 1988). Thus, given this lack of economic investment, Southern communities do not experience the educational benefit of knowledge exchange between diverse communities. This may further increase differences in levels of tolerance toward LGB people between the regions. For example, unlike Southern communities the social environment in the North could encourage greater knowledge and understanding of persons from different cultures with different lifestyles. Increasing knowledge and understanding of sexual and gender minorities may encourage greater tolerance toward LGB tourists, and this might foster greater tolerance toward LGB people from the local community. This interpretation is consistent with contact theory. Contact theory holds that social prejudice can be reduced by introducing a member of one group to another group in certain circumstances, such as when they have a common goal. In the case of tourism the common goal is to have a successful tourist industry that is satisfying for holiday makers and likely build and sustain the local economy. Moreover, perceptions and attitudes of residents toward the impact of tourism are likely to be an important planning and policy consideration for the successful development, marketing and operation of tourism programs (Ap, 1992; Reisinger and Turner, 2011). The acceptance and tolerance of tourists by Northern residents, where much of the tourism industry is based, has been acknowledged as vital for the success of the tourism industry in St. Lucia (Thyne et al., 2007). This suggests that the Northern communities could have grown more tolerant of people whose behaviors and identities deviate from traditional St. Lucian values and norms. Consequently, research is needed to better understand how cultural differences between the Northern and Southern communities might impact St. Lucian LGB people's experiences of intolerance. This is consistent with studies of homophobia in other former British colonies, with homophobia greater in rural regions of Zambia, Australia, and Nigeria for example. Researcher have attributed this geographical patterning to the lack of modernization and educational resources available within rural outer-district communities (Robinson, 2010; Marini, 2011). Rates of depression are also higher within those areas compared to the modernized cities (Morgan, 2008; Marshall, 2012). Therefore, if levels of intolerance are much greater in the South of St. Lucia, Southern LGB people might also experience poorer psychological health than their Northern peers.

Goldberg (2016), for instance, explains that *passing—*purposely suppressing and concealing an LGB identity and performing actions that make others believe that they are heterosexual—is commonplace when LGB people move between social environments that are high and low in their tolerance of non-heterosexuality. It is possible that if there is a Northern vs. Southern difference in levels of tolerance LGB individuals might also experience and report regionalized *passing* in the district that is most intolerant. Goffman (2009) explains that *passing* might have an adaptive function for LGB people in that it helps them avoid homophobic hatred and intolerance. However, there is evidence that it comes with a high social and psychological cost. Goldberg (2016) suggests that the dual identity prevents the person from fully embracing their true authentic self that can induce stress, despondency and dejection. Guss and Drescher (2005) and Remafedi (1987a,b) explain that the anxiety and stress associated with *passing* as heterosexual can often transform into anger that presents as violence, as well as verbal, physical and other negative reactions to friends and family with the strongest negative reaction reserved for parents. The interplay of isolation, fear and anger can also lead some to experience depression, chronic stress, and helplessness (Guss and Drescher, 2005). Therefore, if LGB people are found to practice *passing* between the North and South, it may have some negative impact on their psychological health and well-being. In particular, this could be the case for those living permanently in the South who may feel totally restricted in the expression of their LGB identity.

### Identifying the impact of homophobia on the psychological health and well-being of LGB people in St. Lucia

Studies show that culture must be taken into account when studying the experiences and perceptions of psychological health and well-being of non-white non-western communities (Liamputtong, 2008, 2013). Culture as an organized set of customs, rituals, beliefs, and institutions that shapes peoples cognitions and behaviors to fit its patterns (Müller, 2010) is a quality of all societies. Since the 1980s research has shown that culture can construct peoples understanding of health and well-being, their presentation and perception of symptoms and their understanding and adjustment to treatments (Shaikh, 1985; Chahin, 2009; Fernando, 2014; Roger and Pilgrim, 2014). Consequently, the identification and treatment of psychological illnesses can be complex for people from minority ethnic groups living in nations with a white majority population (Shaikh, 1985; Fernando, 2014; Roger and Pilgrim, 2014). However, there is a paucity of literature about how St. Lucian culture has constructed local peoples understanding and perception of psychological health and well-being. This is problematic for this study given as the cultural concepts and language used to describe conditions such as depression and stress differ from those found in the United States and United Kingdom. For example, Afro-Cubans are more likely to somatize psychological disorders and express the disorder(s) through such cultural idioms as *ataques de nevios—Eng: attack of* nerves. For many Afro-Cubans, *nevios* is an acceptable way of expressing psychological disorders but it is not considered in most standardized diagnostic manuals as its symptoms can be present in a range of psychological disorders (Chahin, 2009). Additionally, when African-American men experience depression they can present symptoms that are different to those presented by other ethnic groups; and particularly irritability and anger can be expressed instead of sadness that it is usually associated with in White-American communities (Alvidrez et al., 2008; Angel and Williams, 2011; Lawman, 2012; Shefer et al., 2012). Therefore, given limited knowledge about health and well-being in St. Lucian culture this study will allow participants to describe the impact of homophobia on their psychological health and well-being using their own local terminology and concepts. The use of Western psychological health tools and measures in this study would also be inappropriate and unethical because they might be antagonistic to the unique beliefs, conceptions, and antecedents of health and well-being in St. Lucia (Shaikh, 1985; Liamputtong, 2008, 2013; Fernando, 2014; Roger and Pilgrim, 2014).

### The present study

Existing literature alludes to the Caribbean as the “most homophobic region of the world” (Padgett, 2006; Rowley, 2011), and past research demonstrates that homophobia in the region has had a profoundly negative impact on the psychological health and well-being of the regions Afro-Caribbean LGB populace (e.g., White et al., 2010). However, few empirical psychological studies have explored (1) sexuality and homophobia related experiences in creolized Caribbean cultures, and (2) possible factors that may make certain Black youth more or less vulnerable to homophobia in St. Lucia and across the wider Caribbean. This study sought to explore these issues by examining perceptions and experiences of homophobia in St Lucia, and how geographical patterning between the North and South of the island might be linked to skin-shade intolerance. Given this, the present study included the perspective of LGB people from the North and South of the Island, and people who ranged in skin shade identity. Additionally, including LGB people who markedly differed in age, socio-economic, and education status served two aims. First, this strategy facilitated the investigation of whether tensions of homophobia were similarly and differently experienced across Black St. Lucians. Second, it facilitated further exploration and theorizing regarding the shared perceptions and experiences of homophobia in St. Lucia. Consequently, thematic analysis was employed to investigate the following question: How do skin color and regional disparities in culture impact experiences of homophobia in St. Lucia?

## Methodology

### Participants

A purposive sample of nine participants (male *n* = 5 and female *n* = 4) who identified as St. Lucian and as Lesbian (*n* = 2), Gay (*n* = 5), and Bisexual (*n* = 2) participated in the study. The participants were aged 18 to 46 years (*M* = 28, *SD* = 9.8) and identified as both St. Lucian citizens and as individuals who had lived in St. Lucia for more than 10 years. The study employed homogenous purposive sampling, a technique used to recruit and study the experiences and perceptions of persons with similar traits or characteristics (Nicholas, 2008). The researchers are cognizant that LGB individuals and communities are not *totally* homogenous. However, in the specific context of this study, the interviewees were considered homogenous as they identified as non-heterosexuals who live in St. Lucia, and thus had relevant knowledge and experience of homophobia or the dislike and hatred of persons attracted to others of the same-sex. In light of the nature of our target population (hidden and hard to track) the lead researcher (JC), as a gay male of St. Lucian background with an existing rapport with the target population, used contacts and networking to recruit participants.

The participants identified as members of the Afro-Caribbean and specifically Black St. Lucian, community. However, in respect of the pigmentocractic structure of St. Lucian society and the nature of this research, participants also provided information about their skin-shade identity. There are over 110 skin-shade identities in St. Lucia that can be categorized broadly into three overarching identifies: white-skinned, light-skinned, and dark-skinned (Gamman, 1994; Chivallon, 2011, Cox, 2002; Malcolm, 2012). Four participants identified as light-skinned, known in *Kwéyòl* as *Chaben* (brown-skinned female) and *Chabin* (brown-skinned male), and five as dark-skinned. Five participants resided in the North of the Island and three in the South, and one participant did not wish to disclose information about their district of residence. The majority of participants also identified as Catholic (*n* = 5) and the remaining four as Rastafarian (*n* = 2), 7 Day Adventist (*n* = 1), and agnostic (*n* = 1).

### Procedure

Following University ethical approval, adverts were placed in LGB media, handouts, and posters to recruit participants. The participants underwent an initial screening with the lead researcher (JC) prior to their participation in the research. If they were suitable for the study they were sent further information to ensure they were aware of all the specificities of the study and their right to withdraw from the study at any time. There was no element of deception within this study and the information sheet provided the participants with all the information necessary for them to make an informed decision about whether to participate. Inclusion criteria required participants to be 18 years of age or older, and to self-identify as LGB and St. Lucian.

On the day of the interview, the participants were required to sign a consent form. The data was collected through face-to-face one-to-one semi-structured interviews. The interviews were conducted in a quiet conference suite of a local business and at a local library, and participants were compensated for their travel. The lead researcher (JC) conducted the interviews in English. To protect the identity of the participants, the research team ascribed pseudonyms to each of the interviewees.

A conversational approach was used throughout, allowing interviewees to contribute toward the direction of the discussion. Broad open questions with prompts (available on request) were used to allow for the emergence of related but unexpected issues. The interview questions were developed following a review of existing literature and were as follows:

- How do would you describe your sexuality and sexual identity?
- Would you consider your sexuality and sexual identity to be an important element of what defines you as a person? And would you be comfortable with the researcher addressing and describing you as lesbian/gay/bisexual in this research?
- Would you begin by describing your experiences as a lesbian/gay/bisexual persons in the St. Lucia? Can you provide some examples?
- Whereabouts do you live on the island? And do you also work in the district in which you reside?
- Have you ever had the experience of working outside the area where you live? If so, what were your experiences?
- If not, what is your perception of working in the different districts?
- What are your thoughts and experiences of homophobia in the North and the South?
- How do you feel about the skin shade and race issues in St. Lucia?
- What are your thoughts on skin shade and sexuality? Has it ever affected your experiences?

All psychological health and well-being related questions were flexible depending on each interviewee's responses. The general protocol for using the follow-up questions was as follows:

- I spotted on question ….…you said “………” would you mind explaining a little more about this?
- Could you explain what you mean when you said “…………”?
- How did that experience make you feel and did it have any impact on you?
- Can you tell me a bit about whether the experience had any impact on your health and well-being?

Each interview lasted ~50 min, and was audio recorded and transcribed. All of the transcripts were checked for wording and grammatical errors, and all personal identifiers were removed. Once complete, the transcripts were then sent to the interviewees for member checking (allowing the interviewees the check the accuracy of transcripts) and the interviewees were given 3 weeks to review the transcripts and report any discrepancies or concerns. All of the interviewees returned the transcripts confirming satisfaction with their accuracy. Although, there was no element of deception within the study, participants were still given a full debrief to ensure that they understood fully the nature of the study and check that they did not want to withdraw their data.

### Analytical strategy

A critical realist thematic analysis was conducted on the interview data, and extracts were used illustratively (Braun and Clarke, 2013). This method was used to illustrate underlying themes within the discourse, and to enrich the understanding of the experiential perspective of homophobia in St. Lucia. “Critical realist thematic analysis” is conceptualized as research that uses thematic analysis from a critical realist stance. This perspective recognizes the centrality of culture and society in constructing the nature of knowledge and perceptions of reality, and theorizes that language characterizes and shapes the meaning of social and interpersonal “worlds.” Thus, critical realist thematic analysis is situated between what Braun and Clarke (2013) describe as the constructionist and essentialist perspective of thematic analysis. This approach helped ground the analysis within the experiences and perceptions of homophobia the participants described whilst producing a critical and contextualized analysis.

In conducting the analysis, we read the data and identified the themes that tended to occur across participant accounts. All three authors (JC, BM, and DW) performed an initial deductive thematic analysis on the interview data (Braun and Clarke, 2013). This method was used to illustrate focused themes within the available texts. In order to achieve this, JC, BM, and DW familiarized themselves with the data by reading and rereading the texts, coding the data, and identifying candidate themes. After revisiting the data again the themes which were identified across participant accounts we clarified, defined, and named. The analysis was undertaken on a latent level, and thus within each theme we paid particular attention to the meaning of participants' accounts, as well as the socio-cultural and relational context in which their perceptions and experiences were situated. However, unlike other forms of latent thematic analysis, the latent aspect of this study is located in the discussion section. This foregrounded interviewee's voices of the interviewees and distinguished these from our interpretations of the meanings behind their perceptions and experiences. The critical realist approach used was also perceived to provide an ethical position that upholds the personified reality of experiencing homophobia whilst recognizing that participant accounts are socio-culturally constructed (Willig, 2010).

### Reflexivity and rigor

The credibility of this study was assessed and validated by immersion and member checking (Onwuegbuzie, 2004; Polit and Beck, 2014). The researchers assigned to undertake the analysis (JC and BM) were immersed in the data by repeatedly reading the transcripts and listening to the audio-recordings. After each data collection session and upon completion of analysis, the lead researcher (JC) performed member checking, and all participants responded favorably. Member checking is a process of gaining feedback from participants on the accuracy on transcripts and research findings. It enables respondents to validate the accuracy of a transcript and enhances credibility, validity, and transferability (Willig, 2010). Dependability (the openness and accountability of the research process) was assessed and supported by collecting records, including minutes from meetings, written analysis, and correspondence between the research team (e.g., email).

Dependability and credibility were also assessed and supported through the process of reflection. The lead researcher was cognizant of his relationship with the other members of the research team and the participants, and how his behavior, during interview sessions, may impact upon the responses of interviewees. The research team spent substantial time reflecting on their own positions in the research and data collection experience. It must be acknowledged that JC, the lead researcher is multiracial (White, Native-Caribbean, and Black Caribbean) and of Caribbean background, whereas DW identifies as white and BM as mixed race (white and black African). Thus, JC has empathy for St. Lucian LGB people and the challenges that they face. To safeguard against bias, JC kept a reflective diary that logged his emotional responses after analyzing transcripts. In doing so, JC also debriefed with the rest of the research team after analyzing emotionally provoking stories. Currently there are no strict guidelines on the measures and checks needed when interpreting data in critical realist epistemology. However, by having three researchers from different ethnic backgrounds involved in the analysis, we were able to enhance the confirmability and credibility of our interpretations through building consensuses we each other (Creswell and Miller, 2000). We did this by discussing the themes, codes and quotes; and when disagreements arose revisions were made until a consensus was reached.

## Results

Two master themes emerged from the analysis: skin-color oriented tolerance, comprised of three sub themes (*light-skin supremacy, acceptance through education, and health consequences*); and North vs. South divide, comprised of two sub-themes (*tolerance and safety and regionalized passing*; see Table 1). Themes and illustrative quotes are presented below.

**Table 1:** Summary table of findings.

**Master themes**	**Sub themes**	**Concept**
Skin-color orientated tolerance^**^	Light-skin supremacy^**^ Acceptance through education^*^ Health consequences^*^	Greater levels of socio-cultural tolerance toward lighter-skinned LGB people. Homophobic abuse and hatred targeted towards dark skinned LGB people.
North vs. South divide^**^	Tolerance and Safety^**^ Regionalized Passing^*^	Greater levels of tolerance toward LGB people in the North. Exposure to homophobic violence and abuse in the South, but not the North.

** *All nine participants endorsed the theme*.

* *Fewer than nine participants endorsed the theme. Specific Sub-Themes: Health Consequences (n = 7), Acceptance through Education (n = 4), and Regionalized Passing (n = 8)*.

### Skin-color oriented tolerance

The participants reported experiencing and observing greater levels of tolerance toward lighter skinned LGB people than their darker skinned peers. As such, dark skinned LGB people reported experiencing greater levels of homophobia and psychological distress and anxiety than their lighter-skinned peers.

### Subtheme: light-skin supremacy

Most interviewees had experienced and witnessed how the shade of LGB individuals' skin-color shaped others tolerance of their LGB sexual orientation. They described how greater tolerance was shown toward lighter-skinned LGB individuals compared to their darker skinned peers. This is exemplified by Jamal, who self-identified as “dark-skinned gay male,” when describing his experience of “coming out” to his friends, family, and local community:

“*As a darker-skinned person, people did not accept me but people seemed to be okay with some of the gay men of high complexion, it certainly felt as though being lighter-skinned meant that it was okay.”*

Interviewees who self-identified as light-skinned also reported experiencing greater levels of tolerance toward their sexuality including when they exhibited gender non-normative behavior. For instance, Jahmal, who self-identifies as a “light-skinned gay male,” explained that his colleagues and friends tolerate his sexuality even when he exhibits feminine behaviors:

“*they …tolerate me more even though I am femme (eng: feminine) the darker gay guys are not treated as well”*

As product of skin color oriented tolerance, the participants explained that it is not acceptable to simultaneously have and embrace the identities of being “dark-skinned” and “LGB.” This signifies what might be skin color oriented identity intersectional issues for some LGB people in St. Lucia. John, a male who self-identified as gay and “dark-skinned,” said:

“*It's just more acceptable to be white or mixed race and gay than black and gay”*

As part of this skin-color oriented tolerance interviewees reported experiencing and witnessing darker skinned LGBs undergoing homophobic bullying, violence, and discrimination that was worse than that experienced by their lighter skinned peers. When asked to explain experiences of this cultural phenomenon, Leyroy, who self-identified as a “dark-skinned gay male,” compared his experiences to those of his lighter skinned LGB friends:

“*lighter skinned friends get no trouble at all, people know they are gay and they get treated with respect. From the way I feel and the experiences I have had, I do sometimes feel that they do no not experience as much suffering.”*

Marionette, 46-year-old self-identified light-skinned Bisexual, also explained that:

“*if I was darker, I think I would have faced a lot more discrimination because of my sexuality”*

As a result of their experiences, the interviewees reported perceiving St. Lucian society and culture as being unaccepting and intolerant of dark-skinned persons engaging in sexual and romantic relationships with same-sex individuals. Rochelle, who self-identified as a “light-skinned Lesbian” described this as follows:

“*I'm a light brown color, whereas, if someone is much darker than me I don't think they will get accepted as a gay person, I just think they stand out a lot more and it is not really seen as acceptable to see them with the same sex”*

The interviewees attributed their experiences of this tolerance to the superior power and socio-occupational privilege held by lighter-skinned persons as the societal elite. Martin, a self-identified light-skinned gay male, summarized this in the following way:

“*I mean light-people we are power, and sometime I feel that people allow me as a gay because my dad's white ……I am gay but they allow me because lighter statuses is seen as more beautiful and rich.”*

Similarly, Zanthe, a self-identified “light-skinned bisexual,” also said that:

“*In this society I live in lighter people like us are still given many advantages even with my sexuality…”*

Many interviewees also described that the social privileges attached to being light-skinned had consequences for how LGB individuals' expressed their sexuality. Some experienced and witnessed dark-skinned LGB individuals undergoing greater socio-cultural pressure to conform to heterosexuality. Marionette, explained that:

“*Society places a lot more pressure on darker people to follow our traditional cultural values, where we were traditionally against homosexuality in all honesty there are a lot of dark-skinned gay people here in St. Lucia, but from what I see they don't get treated as well as lighter gay people like me.”*

A number of interviewees spoke about concealing their sexuality to avoid the racially targeted homophobic tension, discrimination and hostility toward dark-skinned LGB people. This is exemplified by Nathan, who said that:

“*I would say my experiences have been negative as I cannot be myself I have to pretend I am straight to avoid tension.”*

### Sub-theme: acceptance through education

Interviewees reported interpreting differences in the levels of tolerance toward dark-skinned LGB people based on their education level and occupation. For example, before Jamal became a Physician he worked in a retail store and between these occupations he interpreted differences in the way others reacted toward him as a “dark-skinned gay male”:

“*before I was a physician, I worked in clothes stores and it was in those kind of settings that my skin-shade really influenced the way people treated me I find that as a darker skinned people did not accept me but people seemed to be okay with some of the gay men of high complexion.”*

Similar accounts were also recorded by three other interviewees. For example, Priscilla, who self-identified as “dark-skinned Lesbian,” explained that obtaining higher education is a means of climbing what she described as the color caste system. Through education she explained that she was afforded privileges (including others increased acceptance of her non-heterosexuality) which would have otherwise only been available to her light skinned peers. Priscilla said:

“*You can't escape this (intolerance towards dark-skinned LGB persons), education is the only way out, it gives us folks (dark-skinned LGB persons) a chance it improves our social status, it was the only way out for me, people have the highest respect for educated people doctors lawyers black white gay or straight ….”*

### Sub-theme: health consequences

The interviewees interpreted the psychological health outcomes of homophobia in St. Lucia as largely dependent on the skin-shade of the LGB individual, with darker skinned LGB individuals appearing to experience poorer psychological health. One of the most noticeable issues was the depression experienced by dark-skinned LGB people. For example, when asked how their experiences impact on their life, Priscilla explained:

“*my experiences have affected me emotionally it has made me so depressed”*

Others described feeling chronic anxiety and stress, secondary to the day-to-day worries and fears associated with racially targeted homophobic intolerance and hatred of dark-skinned LGB person. Maria, a 32-year-old self-identified dark-skinned Lesbian, described that:

“*This has made me such a nervous person; I worry about every little thing it has made me a social recluse”*

As a result of their experiences and their psychological impact, some self-identified dark-skinned interviewees reported seeking the help of medical professionals. John reported experiencing depression and anxiety as a result of the abuse and discrimination he experienced and explained that:

“*my doctor has actually placed me on anti-depressants.”*

## North south divide

The participants reported a cultural disparity in levels of tolerance toward LGB people between the Northern and Southern region of the Island, with greater levels of tolerance in the North than the South.

### Sub-theme: tolerance and safety

The interviewees experienced what they interpreted as cultural differences in levels of tolerance toward LGB people between the Northern and Southern region of St. Lucia. They described experiencing and witnessing greater levels of socio-cultural tolerance in the North and less in the South. Zanthe, a female who lives in the South but commutes to the North for work purposes, explained that:

“*In the South they are much more outdated, so it is not a great place for gay people, I just find that in the North they are a lot more open minded people are a lot more tolerant up North.”*

Interviewees also reported experiencing and witnessing greater levels of homophobic violence and discrimination in the South compared to the North. For instance, Jamal described his experiences:

“*last year we sent a male nurse on a home visit to Laborie, he was attacked by the sons of the elderly male patient” for the reason of homosexual suspicion, the “fact that he is Male and a Nurse made the guys suspect that he was Gay,”* even though he is married heterosexual.

The interviewees described the North as a popular destination for tourists and as the center of St. Lucian tourism. They interpreted tourism as contributing positively toward the greater level of tolerance they experienced within the Northern region. They explained that Northerners are raised in an environment of greater diversity than their Southern peers allowing them to interact socially with those of different religions, sexualities, and ethnicities. Consequently, many interviewees explained that this led those in the North to have “better” understanding and tolerance of differences in sexuality. When asked to elaborate on her experiences between the North and South, Priscilla explained that:

“*there are a lot of tourists in the North, so people in those areas are taught to be tolerant because if they discriminate it can destroy our tourism industry which we thrive on here in St. Lucia”*

The issue of tourism was further exemplified by Marionette:

“*They are more modern and there are a lot of tourists when I am in that environment there are a lot of different people so locals are naturally more tolerant”*

Through interacting with LGB people, the interviewees explained that Northerners bettered their intellectual understanding of sexuality that improved their levels of tolerance. Jamal, a gay male who spent his childhood growing up in the South but now lives in the North, explained that:

“*We have a lot of holiday makers (persons of vacation) in the area, so most of my co-workers have learnt to be tolerant of all people who are different…”*

Interviewees also reported experiencing what they interpreted as differences in individuals' understanding of same-sex relationships between the North and the South. They described Southern communities as perceiving same-sex relationships and homosexuality as life choices that individuals can control. When asked to explain why living in the North makes him happier, Leyroy explained:

“*People are educated up here, most of the people around me are intelligent people, these are people that know I can't change the way I am…”*

The interviewees also linked Southern intolerance toward LGB people with the lack of educational resources available in Southern communities. Some interviewees perceived a lack of education as leading to specific lay theories of sexuality that encouraged Southern intolerance:

“*they are poor they can't afford to be educated, and they think being Gay is an illness or a curse.” (Maria, a resident of North of the Island)*

“*Most country people are not educated, that's why I think I had such bad experiences” (Marionette, a resident of North of the Island)*

Stress, anxiety, and safety were issues also vocalized by the participants. In light of experiencing and witnessing greater levels of intolerance in the South, interviewees reported feeling safer in Northern towns and villages on the Island, and feeling stressed, scared, and anxious when in Southern towns. Martin who works in the North, but commutes to the South for work purposes, explained that*:*

“*I feel much safer in the North, people treat gay people with respect here, they are much more acceptant of everyone who is different, it is the only place on the island where I feel safe to work without having to look over my shoulder all the time”*

The issue of personal safety is further exemplified by John:

“*Because I felt safer up North commuting meant that I was heading out of my comfort zone, working in the North I feel happier and safer here people are more acceptant”*

### Sub-theme: regionalized passing

Interviewees described altering how they presented their sexual identity when commuting between the Northern and Southern districts. When in the South interviewees reported presenting their sexual identity to others as heterosexual. When asked about his experiences of traveling between the North and the South, one participant explained:

“*When your cross Castries into those area, it is like you going back in time so that is where I have to jump between who I am and who they want me to be, It's kind of odd.” When asked what he meant by “who I am and who they want me to be” he replied* “I have to act like a straight man for my own safety.”

Interviewees also described how they decided to alter how they presented their sexuality when commuting from the Southern to the Northern region, shifting from a heterosexual identity back to their “true” LGB identity in both social and occupational settings. In the psychology of human sexuality, this type of identity shifting is known as *passing*. When asked “why” they regionally shift their sexual identity, interviewees explained that they felt safer in the Northern region and thus able express their true sexual orientation more openly in the North of the Island. One participant explained:

“*I feel safest in Gros Islet and Rodney Bay, only because they are more modern and there are a lot of tourists, when I am in that environment there are a lot of different people so locals are naturally more tolerant.”*

Thus, for the interviewees, shifting their public sexual identity was a protective strategy against anticipated homophobia, discrimination and abuse. Maria, who lives in the North and commutes to the South, explained:

“*I used to act I had a husband as I know what people are like around there,”*

When asked what she means by “what people are like around there,” she explained that

“*its even more risky to be gay in the South than it is up North, as the people are just far too behind I just know they would beat me.”*

Although, interviewees openly expressed their LGB identity in the North, all interviewees revealed that they also conceal their sexual orientation when at work to prevent what they perceived as job based discrimination based on her sexuality. Zanthe, said:

“*Well in front of my co-workers it depends on who I am with, that may make me sound fake but I don't want my sexuality to cost me my job”*

All of the interviewees reported feeling anxious and distress every time they had to conceal their “true” sexual identity in the South. For many interviewees shifting of sexual identity meant that they could not be their true inner self and that this concealment induced feelings of depression. The participants explained that their experiences of anxiety stemmed from day-to-day worry about losing their job or relationship with friends and family in the South. This was worsened by being worried that their deception of concealing their sexuality would be discovered:

“*I have to pretend I am something I am not, It makes me feel miserable when I cannot be myself.”*—says Nathan.

“*I endlessly worry that people will find out my sexuality and think bad of me, so I tend to do a lot of things to prevent people finding out”*—says Martin.

## Discussion

This is one of the first qualitative studies exploring perceptions and experiences of homophobia amongst LGB individuals in St. Lucia, West Indies. The interviewees raised a number of serious concerns related to social and health issues. Their accounts suggest sexuality related stigma affects negatively the lives of LGB people in St. Lucia. One of the most noticeable similarities across participants' accounts is how the shade of LGB individuals' skin-color shapes others' tolerance of their sexual orientation. Participants described their experiences of skin-color oriented tolerance as fueled by the power of superiority held by light-skinned persons as the societal-elite. These experiences illustrate what might be the existence of a skin-color oriented hierarchy of tolerance toward LGB people. This could exist coherently alongside, and be reinforced by, the pigmentocractic structure of St. Lucian society (Gabriel, 2007; Glenn, 2009). Additionally, given the pigmentocracy is widespread across the Caribbean (Tate, 2015, 2016) this variation in perceptions and experiences of homophobia may not be unique to St. Lucia. Therefore, it is important to address this issue in research on other societies in the region.

Two possible conclusions that can be drawn from our findings: anyone who is lighter-skinned is treated better in St. Lucian Society regardless of sexuality, or being lighter-skinned is equated with being white and possessing cognitions and behaviors culturally associated with and ascribed to *white people*. Therefore, could being LGB be seen as expected for white and light skinned people? Other studies have shown that within many Afro-Caribbean and African-American communities, homosexuality is perceived as something belonging to the white race and unnatural for black persons (e.g., Ford, 2008). Consequently, it is likely that being LGB in St. Lucia is perceived as natural for white and light-skinned persons but unexpected for darker-skinned persons. This could fuel intolerance toward darker-skinned LGB people and greater tolerance may be another privilege of being “light-skinned” in St. Lucia.

Issues of intersectionality (Rodarte-Luna, 2008) are evident in interviewee's accounts. Given the colonial history and pigmentocractic structure of St. Lucian society, racial identities in St. Lucia extend beyond broad rigid categories of Black, White, and Asian (etc.) to skin-shade identities (Gamman, 1994; Wilton, 2005; Hall, 2008; Carter, 2013) and in St. Lucia these are white-skinned, light-skinned and dark-skinned (Gamman, 1994; Cox, 2002; Chivallon, 2011; Malcolm, 2012). Participants' accounts suggest that being “dark-skinned” and “lesbian, gay, or bisexual” is unacceptable in St Lucian culture. These findings support existing literature that skin-shade identity is largely perceived as *superior* to most other identities, and a primary identity from which all other identities and behavioral expectations are structured (e.g., Kruijt, 2005; Ford, 2008, 2013; Rosario, 2011; St-Hilaire, 2011; Shirk and Edmond-Poli, 2012; Flynn, 2012). This sexuality and race nexus might challenge some dark-skinned LGB people to demonstrate the authenticity of their ethno-racial identity because of their non-heterosexuality. Research demonstrates that in most instances Afro-Caribbean heterosexuals perceive Afro-Caribbean LGB persons as “selling out” to what is often referred to as the “white disease” (homosexuality). Thus, Afro-Caribbean LGB individuals often report experiencing being labeled as an “*Oreo*” and/or “*Coconut*” (Das Nair and Thomas, 2012. p. 97)—black on the outside, but “white” on the inside—to shame them for not prioritizing their racial identity and its associated behaviors (Das Nair and Thomas, 2012). For many Afro-Caribbean LGB people this form of shaming indicates that their community is not accepting of people who attempt to embrace both identities simultaneously (McKeown et al., 2010). For instance, in a study by McKeown et al. (2010), an interviewee stated:

“*I have a Black female friend who…complained that I was focusing too much on being gay and not enough on being black. It is assumed by her that the black identity is superior and not complementary, and the implication in her voice and manner suggested that I was ‘letting the side down’. She and other associated seem to struggle with the idea that you can be both simultaneously.”* (p. 5).

Therefore, what does this mean for the development of healthy skin shade and sexual identities and experiences of psychological well-being in St. Lucia? Given the limited literature, it is reasonable to suggest that some dark-skinned LGB persons may experience a cognitive dissonance between the two identities of being dark-skinned and LGB. Opposing meanings and behavioral expectations ascribed to these two different identities (e.g., dark skinned = not homosexual) could elicit social, cultural, and psychological struggles when individuals attempt to fuse and embrace these two identities as was apparent in experiences of some of the interviewees in this study. This interpretation is also consistent with the work of Bhugra (1997) who documented the importance of racial identity in South Asian minorities in the UK and its impact on sexual identification and expression. He found also found a cognitive dissonance between individual's ethno-racial and sexual identity. Many South Asian ethno-racial identities emphasize the importance of family and religion, and many of the LGB people in his study perceived this as clashing with their LGB identity. Due to this cognitive dissonance, many South Asian LGB people adopted a *false* heterosexual identity around members of their ethno-cultural group and shifted to an LGB identity around other LGB people and members of more tolerant ethno-cultural groups. Additionally, some went beyond adopting a false identity to consciously adopting a double life: *heterosexual* around members of their own ethno-racial group (e.g., getting married to a member of the opposite-sex and having a family), *and LGB* that is hidden from their ethno-racial group. Similarly, participants in our sample made associations between intolerance toward dark-skinned LGB people and concealment of their sexual identity to protect against both suspicion of being homosexual and from being the victim of homophobic violence and abuse from their community. Disconcertingly, over the past decade research has found that prolonged concealment and shifting of a person's *true* identify can often induce chronic stress and mild depression, secondary to the suppression of the *true self* (Nance, 2008).

In that case, are some dark-skinned LGB people more likely to develop unhealthy racial and sexual identities? The literature on the impact of skin color oriented tolerance on experiences of racial and sexual identity in St. Lucian society and culture is limited and requires further research. Nevertheless, it is possible that some dark-skinned LGB persons may develop healthy racial and LGB identities and psychological health in spite of skin color oriented tolerance when protective factors such as resilience and coping behavior are considered. For example, LGB individuals could vary in their use of prescribed western-centric labels of sexuality as a coping behavior. In a society where there is stigma toward dark-skinned persons who identify as LGB, some dark-skinned individuals attracted to the same-sex may dissociate their sexual practice from an identity they perceive socially stigmatized along with the Caribbean's growing *gay scene* (e.g., Human Rights Watch, 2004). Such a dissociation does not always mean that a person, whether dark or light skinned, has an unhealthy identity (e.g., Wellings et al., 2012). In western society it is largely thought that to obtain a healthy sexual and romantic subject-hood people need to subscribe to prescribed labels of sexuality e.g., lesbian and gay (Das Nair and Thomas, 2012). However, according to the work of Asthana and Oostvogels (2001) the use of rigid labels such as Gay, Lesbian, and Bisexual is not common in the non-western societies, except among educated urban persons exposed to the western LGB scene. Even in societies where attitudes toward LGB persons have grown to be more positive, not all persons with sexual and romantic feelings toward the same-sex will identify as LGB (Wellings et al., 2012).

Previous literature highlights a range of cultural, educational and political disparities between the Northern and Southern region of St. Lucia; and some previously unexplored sexualities-related disparities were evident in our findings and in particular greater tolerance toward LGB people in the North than in the South. Previous studies have shown that rates of depression, stress, and suicidality are higher amongst LGB people who reside in communities where levels of homophobia are greater (Morgan, 2008; Marshall, 2012). Given the lack of tolerance in the South, it is likely that Southern LGB people may experience poorer psychological health than their Northern peers but this remains relatively unexplored.

Given the greater level of intolerance toward LGB people in the South, *passing* in the South was prevalent in the accounts of participants. *Passing* appears to be a means of protection against sexuality-related discrimination, attacks and hatred in the South. While occasional *passing* might not be harmful, studies have shown that the frequent and prolonged *passing* can have a negative influence on the psychological health and well-being of LGB people (Risdon, 2003; Harris, 2008; Nadal, 2011). For instance, some studies suggest it can induce psychological distress and depression that is secondary to the affects resulting from suppression of the *true self* (Shelly-Sireci, 2012). Therefore, even as a protective mechanism, *passing* might have a range of adverse consequences that negatively distort the cultural and psychological well-being of St. Lucian LGB people. This was also apparent in experiences of the participants, many whom reported feeling unhappy, saddened, distressed, and depressed when having to conceal their true sexual identity. Additionally, for those LGB people who live and work in the South, rates of depression might be significantly higher as unlike their Northern peers, Southern LGB people may be more restricted in their ability express their *true* sexual identity, desires and feelings.

Further research could focus on the psychological health implications of skin color oriented tolerance. Specifically, we recommend more research on the consequences of self-loathing amongst dark-skinned LGB people. Beyond racial self-identification in St. Lucia, light-skinned people have more education and higher occupational status and privilege than darker-skinned do. Thus, unlike their dark-skinned heterosexual peers, dark-skinned LGB people in St. Lucia might be faced with the dual challenges and stigmas of skin-color targeted homophobia and general skin-color oriented socio-occupational disadvantage and discrimination. Very few other people in society have to endure such stigmas simultaneously and to such an extent. Within such a societal framework, dark-skinned LGB people may experience issues of color self-loathing. We use the term “color self-loathing” to describe individuals who are happy and satisfied with their own broader racial-identity (e.g., Black or Asian etc.) but who have negative feelings toward their own skin-shade and skin-shade identity within their racial-group. This term is not to be confused with “racial self-loathing” or negative feelings toward oneself because of belonging to a specific racial group (Hall, 2008). Color self-loathing is an especially well-known and much discussed issue in Jamaica, a place suffering from an epidemic of skin bleaching (Kovaleski, 1999; Charles, 2003; Pierre, 2013). Clinical studies found a link between dark-skinned disadvantage, skin bleaching and the high rates of depression and substance misuse amongst the region's darker-skinned community (Hall, 2010, 2008; David, 2013a,b). However, given the lack of research on LGB people in the Caribbean region, the issue of color self-loathing within the LGB community still remains relatively unexplored.

The findings also point to a need for additional research on the role of education on sexuality and homophobia in St. Lucia. Other studies have suggested that educational strategies, including schooling reform and educational campaigns, are important in reducing homophobia (e.g., Herek, 1984; Eichstedt, 1996; Black et al., 1999). While there is a lack of research investigating the effectiveness of these interventions in St. Lucia, psychologists have long presented findings in support of this proposition (e.g., Serdahely and Ziemba, 1984; Ben-Ari, 1998; Black et al., 1999). These studies suggest that increases in tolerance toward dark-skinned LGB people and LGB people in the South is possible. However, more research on education and sexuality in St. Lucia could improve our understanding of these issues, and inform culturally appropriate schooling reforms and educational campaigns specifically for St. Lucian society. Schooling differs between societies and cognitive styles differ between cultural groups (e.g., the way people receive, process, and make meaning of social and environmental information). Therefore, educational intervention strategies need to be culturally specific to change cultural attitudes (Ford et al., 1996; Faiola and Matei, 2005; Western, 2005).

## Limitations

Although, the findings of this study provide insights into the concepts of what it means to be “dark skinned” vs. “light skinned,” it has limitations. The interviews were retrospective and memory played a critical role in the accounts shared by the interviewees. Although what interviewees remember is important to the meaning they attribute to events they experienced, the researchers cannot be certain of the veracity of these accounts. The gender and racial identity of the lead researcher who conducted the interviews could have influenced the findings. The lead researcher (JC) is a light-skinned male who resides in the North of St. Lucia and participants may have disclosed different information if they were interviewed by a person of the same gender and skin shade identity as themselves or by a person who lived in the same district (see Liamputtong, 2008, 2013). Thus, replications of this study using participant and researcher gender and skin-shade identity matching during the interview process are encouraged. These replications could provide further evidence of the reliability of the findings and information about the role of skin-shade and gender identity in researcher and participant interactions in St. Lucian culture. Furthermore, the lead researcher used their social contacts within the St. Lucian LGB communities to recruit the sample that could bias the findings (e.g., the sample being more homogeneous than the larger target population, and the lead researcher being middle class and university educated). However, in reality it is difficult to recruit a large and diverse sample of St. Lucian LGB people. Therefore, replications of this study using different sampling methods are encouraged to provide more evidence for the degree of generalizability of the findings. Nevertheless, given the ethno-cultural diversity of the research group we were able to draw on a variety of different perspectives to produce a more nuanced account and understanding of the life circumstances of St. Lucian LGB people. Finally, although the study is exploratory and its conclusions tentative they are consistent with previous research that links negative social experiences to stress, depression and other forms of psychological distress.

## Conclusion

Historically, researchers have neglected the lives of non-white LGB people (Anderson, 2009, 2011; Fisher, 2012) and very few researchers have examined specifically the experiences of Afro-Caribbean sexual minority persons based on their race, ethnicity and sexual orientation. The current study aimed to increase our knowledge of these issues by exploring the experiences of homophobia in St. Lucia. Our study revealed important issues experienced by St. Lucia LGB people: the shade of LGB individuals' skin-color shaped others' tolerance of their sexual orientation, and regionalized disparities exist in the level of tolerance toward LGB people. However, further studies should expand on the LGB people who were not represented in our study, including white LGB people (and LGB people from other racial backgrounds). Additionally, other non-heterosexual individuals who have experienced homophobia unreported in this study, such as those with a pansexual and asexual identity, might provide further insight into the socio-psychological experiences of homophobia in St. Lucia.

## Ethics statement

The Institute of Health and Society reviewed and approved the procedures for this study. Participants read an information sheet and provided their full consent to participate in this study.

## Author contributions

JC developed the study concept and method. JC performed the data collection and analysis and all authors discussed the results. JC wrote the first draft of the manuscript and BM and DW provided comments and revised the manuscript.

### Conflict of interest statement

The authors declare that the research was conducted in the absence of any commercial or financial relationships that could be construed as a potential conflict of interest. The reviewer DMY and handling Editor declared their shared affiliation, and the handling Editor states that the process nevertheless met the standards of a fair and objective review.
